# DNA Abasic Site-Selective Enhancement of Sanguinarine Fluorescence with a Large Emission Shift

**DOI:** 10.1371/journal.pone.0048251

**Published:** 2012-11-20

**Authors:** Fei Wu, Yanwei Sun, Yong Shao, Shujuan Xu, Guiying Liu, Jian Peng, Lingling Liu

**Affiliations:** 1 Institute of Physical Chemistry, Zhejiang Normal University, Jinhua, Zhejiang, People's Republic of China; 2 Chuyang Honors College, Zhejiang Normal University, Jinhua, Zhejiang, People's Republic of China; US Naval Reseach Laboratory, United States of America

## Abstract

Small molecules that can specifically bind to a DNA abasic site (AP site) have received much attention due to their importance in DNA lesion identification, drug discovery, and sensor design. Herein, the AP site binding behavior of sanguinarine (SG), a natural alkaloid, was investigated. In aqueous solution, SG has a short-wavelength alkanolamine emission band and a long-wavelength iminium emission band. At pH 8.3, SG experiences a fluorescence quenching for both bands upon binding to fully matched DNAs without the AP site, while the presence of the AP site induces a strong SG binding and the observed fluorescence enhancement for the iminium band are highly dependent on the nucleobases flanking the AP site, while the alkanolamine band is always quenched. The bases opposite the AP site also exert some modifications on the SG's emission behavior. It was found that the observed quenching for DNAs with Gs and Cs flanking the AP site is most likely caused by electron transfer between the AP site-bound excited-state SG and the nearby Gs. However, the flanking As and Ts that are not easily oxidized favor the enhanced emission. This AP site-selective enhancement of SG fluorescence accompanies a band conversion in the dominate emission from the alkanolamine to iminium band thus with a large emission shift of about 170 nm. Absorption spectra, steady-state and transient-state fluorescence, DNA melting, and electrolyte experiments confirm that the AP site binding of SG occurs and the stacking interaction with the nearby base pairs is likely to prevent the converted SG iminium form from contacting with water that is thus emissive when the AP site neighbors are bases other than guanines. We expect that this fluorophore would be developed as a promising AP site binder having a large emission shift.

## Introduction

Exertion of cell functions is critically dependent on genome fidelity, which is threatened by various DNA lesions [1]–[3]. Abasic site (AP site) is one of commonly observed DNA mutagenic and carcinogenic lesions [4], arising from spontaneous depurination or depyrimidination during in vivo base excision repair (BER) of damaged DNA bases [5]. Therefore, recognition of the AP site holds great promise for diagnostic and therapeutic applications [6].

Although the AP site can be targeted by non-fluorescent small molecules including binder/insertor heterodimer [5], [7]–[9], metalloinsertor [6], [10], redox probe [11], nitroxide spin label [12], and DNA base analog [13], fluorescent small molecules have received much attention due to simplicity and cost saving in the detection technologies. In this aspect, some fluorophores were found to be effective such as environment polarity-sensitive naphthalene derivative [14] as well as the organic probes possessing hydrogen bond moieties that are complementary to the bases opposite the AP site, including naphthyridine [15], [16], pyrazine [17], lumazine [18], pteridine [19], [20] and flavin [21] derivatives. However, due to formation of the static DNA complexes, excited state electron transfer, and the other intricate processes, fluorescence quenching was usually observed [15]–[21]. More seriously, the presence of the AP site-containing DNA (AP-DNA) did not alter the fluorophores' emission wavelength. Thus, high background emissions can not be overcome using these probes as the AP site binders.

We have being focused on seeking new fluorophores exhibiting novel optical properties upon binding to the AP site. A new long-wavelength emission band arising from an excited-state intramolecular proton transfer (ESIPT) probe [22], fisetin, one of natural 3-hydroxyflavonols, was observed in the presence of the AP site. Recently, we found that berberine [23], one of natural isoquinoline alkaloids, can selectively bind to the AP site with a sequence-dependent manner. Although fluorescence enhancement was observed for these fluorophores, the alteration in their emission wavelengths upon binding to the AP site was not more than 60 nm. Herein, another alkaloid, sanguinarine (SG), was employed to achieve a much larger emission shift up to 170 nm when binding to the AP site.

**Figure 1:**
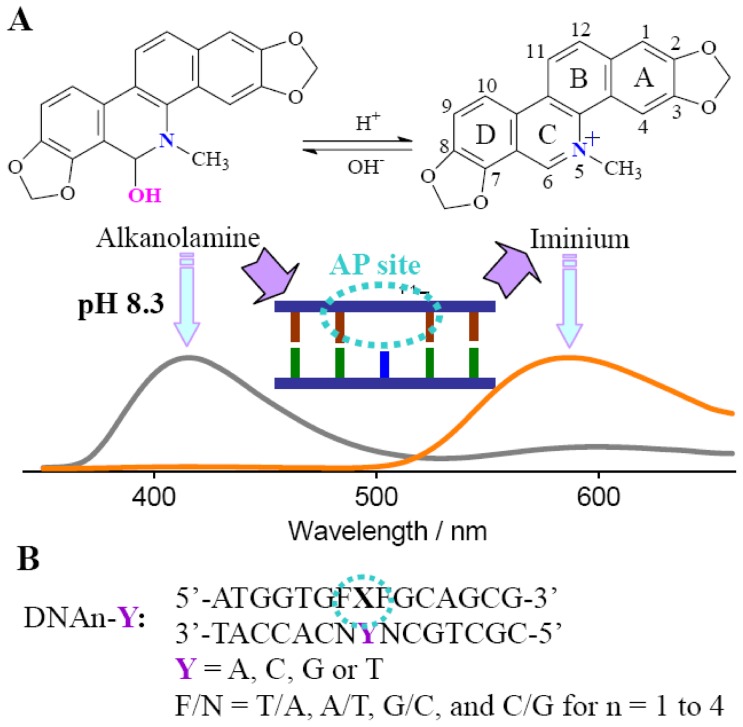
Structures of SG and DNA sequences. (A) SG at the variable forms and schematic representation of the AP site-targeted association of SG with a large emission shift. (B) For AP site-containing DNAs, X = AP site (dSpacer, tetrahydrofuran residue) that is opposed by base Y and flanked by base Fs. Fully matched DNAs (FM-DNA) with X/Y = A/T, C/G, G/C, and T/A were used as controls.

SG belongs to a benzophenanthridine alkaloid, which is known for its antitumor property and possesses the potential for selective/preferential elimination of cancer cells [24]–[28]. Besides its interaction with proteins [29] and amino acids [30], one of the potential antitumor activities of SG is believed to result from its highly specific binding to many types of nucleic acid structures and subsequent modification of the genetic information [31]. SG exhibits a pH-dependent structure equilibrium in aqueous solution between the positively charged iminium (in the pH rang 1.0–6.0) and the neutral alkanolamine (in the pH rang 8.5–11.0) forms (Figure 1) [31], [32]. The iminium form is unsaturated and completely planar, while the alkanolamine form has a buckled structure. SG can interact with polymorphic nucleic acid structures including DNA (B form [33], Z form, triplex [34], quadruplex [35]) and RNA (for example, poly(A) [36]). It is widely believed that the iminium form is mainly responsible for the DNA binding [37]. In addition, binding-induced fluorescence quenching and a strong GC base pair binding preference were observed [38]–[41]. In this work, we found that SG exhibits a sequence-dependent AP site binding behavior in the aspect of the enhanced emission for the iminium form that is converted from the alkanolamine form. Thus, targeting the AP site with a larger emission shift can be realized by thorough conversion of the alkanolamine emission band to the iminium emission band. The mechanism of the sequence-dependent fluorescence behavior is discussed.

### Experimental section

DNA species (Figure 1) were synthesized by TaKaRa Biotechnology Co., Ltd (Dalian, China) and purified by HPLC. The DNA concentrations were measured by UV absorbance at 260 nm using extinction coefficients calculated by the nearest neighbor analysis. Tetrahydrofuran residue was used as the chemically stable abasic site (AP site) for replacement of the naturally-occurred unstable deoxyribose structure. To prepare DNA duplex solutions, the probe and target strands were mixed in equimolar amounts and annealed in a thermocycler (first at 92°C, then cooled down to room temperature slowly) in 0.1 M phosphate buffer (pH 7.0) containing 1 mM EDTA.

Sanguinarine (SG, Sigma Chemical Co., St. Louis, USA) was added to the duplex DNA solution to an appropriate molar ratio at 0.1 M phosphate buffer (pH 7.0) containing 1 mM EDTA. After mixing, the solution was incubated for 15 minutes with gentle stirring. The resulting solution was examined at room temperature within 2 h. Nanopure water (18.2 mΩ; Millipore Co., USA) was used in all experiments. Fluorescence spectra were acquired with a FLSP920 spectrofluorometer (Edinburgh Instruments Ltd., UK) at 18±1°C, equipped with a temperature- controlled circulator (Julabo, Germany). Time-resolved fluorescence decays were recorded on a time-correlated single photon counting FLSP920 system, with excitation at 375 nm. A ludox solution was used as the scatter for the instrument response. The data were fitted with a multiexponential decay and χ^2^ was less than 1.15. UV/Vis absorption spectra and melting temperatures (T_m_) were determined with a UV2550 spectrophotometer (Shimadzu Corp., Japan), equipped with an accessory of TMSPC-8 T_m_ analysis system which can simultaneously control the chamber temperature and detect up to 8 samples by a micro multi-cell.

## Results and Discussion

**Figure 2:**
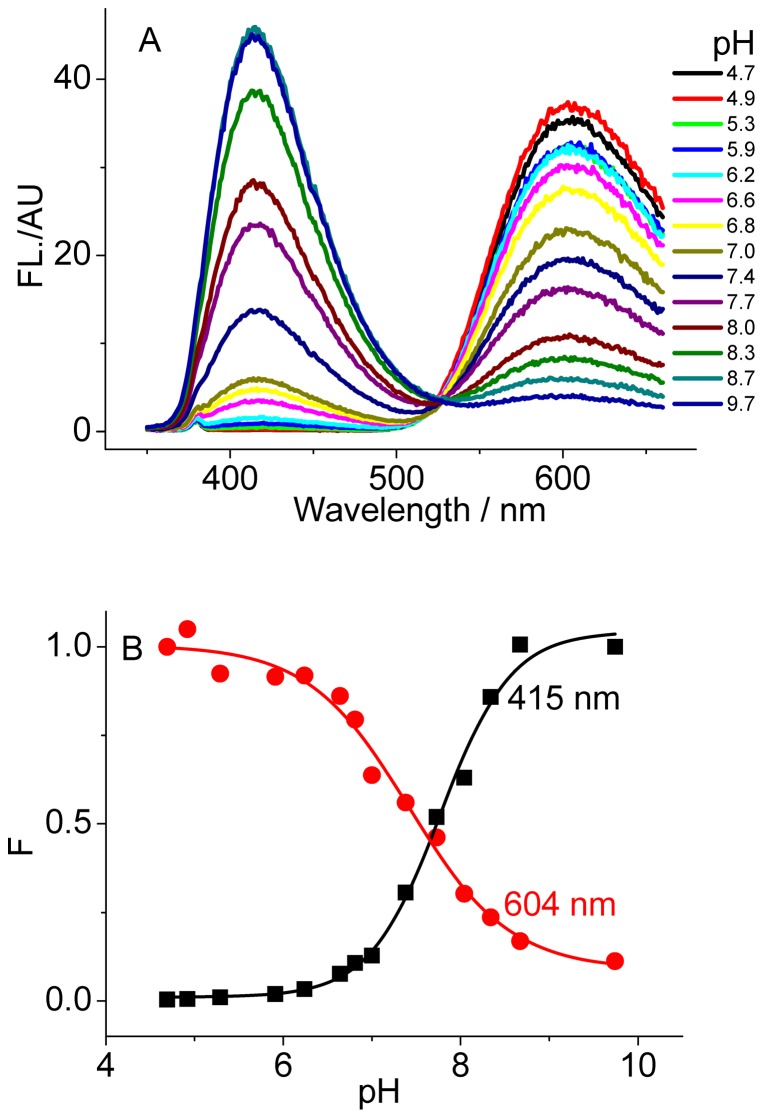
pH dependence of SG fluorescence. (A) emission spectra and (B) the relative intensity alterations of SG (5 µM). λ_ex_ = 336 nm.

In aqueous solution, SG exists in the forms of iminium and alkanolamine and their population is dependent on pH (Figure 1). As shown in Figure 2, the 415 nm emission band increases with the solution pH increasing, while the 604 nm band simultaneously deceases under excitation at 336 nm. Thus, the iminium and alkanolamine forms emit at 604 and 415 nm, respectively [31]. The fitted equilibrium constant *pK*_a_ is about 7.7, which is in good agreement with the previously reported value [32]. The alkanolamine form is not further deprotonated when the solution pH is lower than 11 [42]. According to the absorbance of the two forms at the corresponding extremely low and high pH and the reported fluorescence quantum yield [32], the fluorescence quantum yields of 0.003 for the iminium form and of 0.11 for the alkanolamine form at 604 and 415 nm were roughly estimated with excitation at 336 nm. Importantly, converting between the iminium and alkanolamine forms is reversible vis pH adjustment. This provides us a chance to investigate novel SG-involved applications in biosensing with a large emission shift if it is capable of converting one of the forms to the other upon binding to the DNA targets of interest.

**Figure 3:**
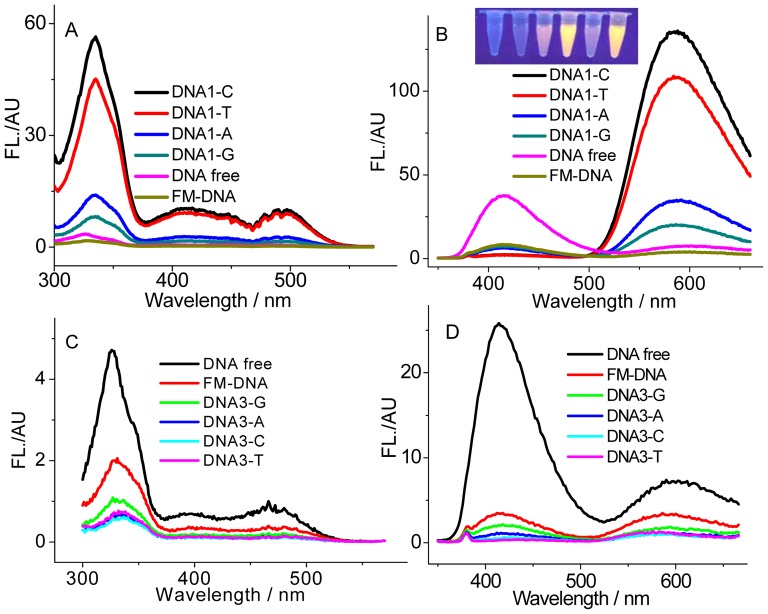
AP site-dependent fluorescence behaviors of SG. Excitation (A and C, measured at 586 nm) and emission (B and D, excited at 336 nm) spectra of SG (5 µM) in the absence and presence of 5 µM DNA1-Ys (A and B) and DNA3-Ys (C and D). The corresponding fully matched DNAs (FM-DNA) were used as controls. Inset: the photographs of SG in the absence and presence of 5 µM FM, DNA1-A, DNA1-C, DNA1-G and DNA1-T (from left to right) under UV illumination.

A DNA binding event usually favored a fluorescence quenching response of the originally populated SG form [31]. Additionally, a conversion of the alkanolamine form to the iminium form was incidentally observed in the presence of a large amount of DNA [33]. We attempted to achieve this conversion but with a large emission shift using a DNA containing an abasic site (AP site) that serves as the SG binding site (Figure 1). The AP site is produced in living cells by loss of a nucleobase and thus surrounded by an unpaired and two flanking bases, in which the hydrophobic microenvironment would be different from the DNA groove regions. A 0.1 phosphate buffer with pH 8.3 was employed here. At this pH, SG presents mainly in the alkanolamine form and DNA is still stable in the B-form. As shown in Figure 3A and B, addition of a fully matched DNA (FM-DNA) induces a heavy quenching of SG fluorescence at both the primarily dominated 415 nm alkanolamine band and even the weak 604 nm iminium band. This phenomenon is highly coincident with the previously reported results [31]. However, the presence of the AP-DNAs with thymines flanking and Y (Y = C, T, A, G) opposite the AP site (DNA1-Ys, Figure 3A and B) makes the maximum of the excitation band between 300 and 380 nm red shift to 336 nm and of the 604 nm iminium emission band blue shift to 586 nm. More importantly, DNA1-Ys strongly quench the SG alkanolamine emission band and sharply enhance the iminium band. Additionally, this enhancement is strongly dependent on the unpaired base Y. The emission intensities for the iminium band in the presence of DNA1-C, DNA1-T, DNA1-A, and DNA1-G are roughly 20, 15, 5, and 3 times higher than that for SG alone. Namely, the unpaired pyrimidines opposite the AP site induce a larger enhancement in fluorescence than the unpaired purines. Distinguishing the AP site binding from the FM-DNA binding can be also easily achieved by the naked eye under UV illumination (much brighter yellow emissions for DNA1-Ys, Inset of Figure 3B). Clearly, conversion of the alkanolamine form to the emissive iminium form indeed occurs in the presence of the DNA1-Ys. Note that the difference in the AP-DNA structures from the FM-DNA is only the AP site. Thus, the binding site of SG in the AP-DNAs responsible for the fluorescence enhancement must be the AP site involved. The optical properties of SG bound in the AP site environment should be different from that directly in aqueous solution. For example, based on the absorbance and fluorescence of SG at the enough high AP-DNA concentration (to make sure that SG is completely associated to the AP site), we estimated that the quantum yield of SG binding to DNA1-C increased to about 0.03, ten times higher than that for SG alone in aqueous solution.

The similar fluorescence enhancement behavior was observed for DNA2-Ys with adenines flanking the AP site (Figure S1). Therefore, at pH 8.3, the presence of DNA1-Ys and DNA2-Ys bathochromically shifts the main alkanolamine emission band of SG at 415 nm to the 586 nm iminium band. Thus, a large emission shift up to 170 nm accompanied by an enhancement in intensity is achieved for SG in targeting the AP site.

Nevertheless, this performance has not been realized for the previously used fluorophores [15]–[23]. On the other hand, fluorescence quenching even to a greater degree than the corresponding FM-DNA was observed when the flanking sequences were changed to guanines (DNA3-Ys, Figure 3C and D). Similarly, such the more seriously quenching phenomenon also occurred for DNA4-Ys with cytosines flanking the AP site (Figure S1).

**Figure 4:**
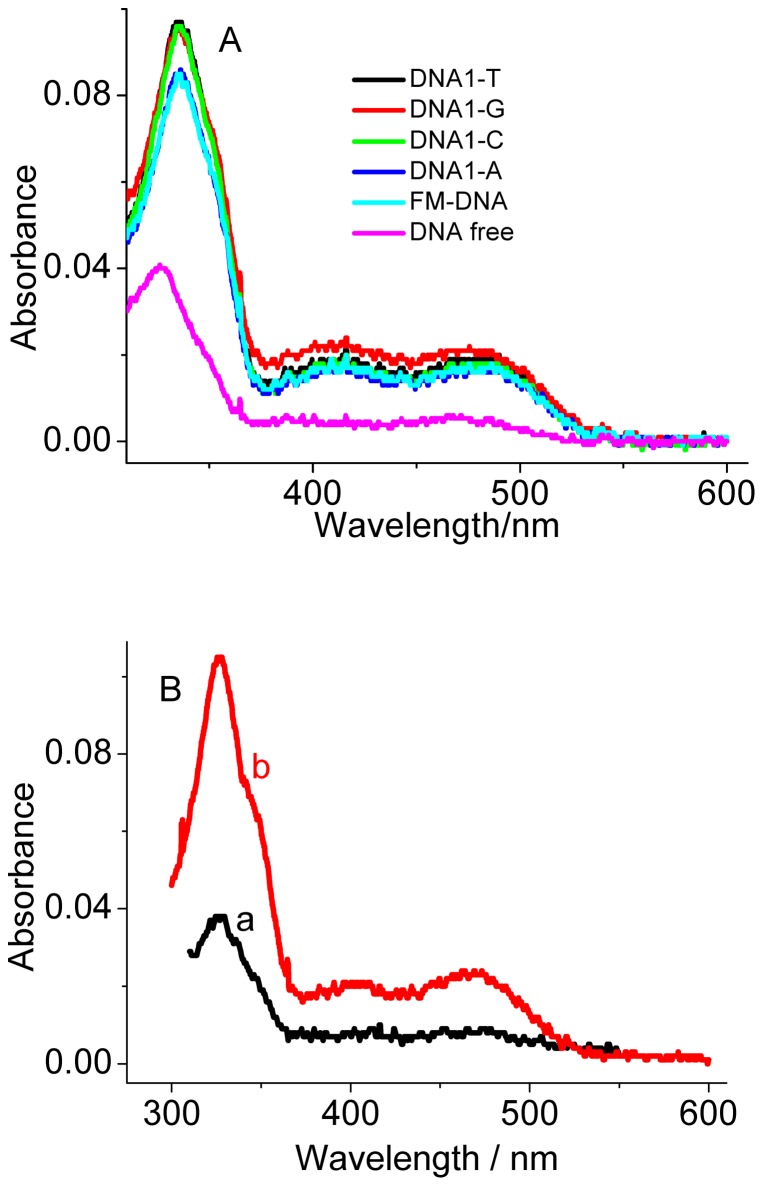
Absorption spectra of SG. (A) UV-Vis absorption spectra of SG (5 µM) in the absence and presence of 5 µM DNA1-Ys. The corresponding fully matched DNAs (FM-DNA) were used as controls. (B) UV-Vis absorption spectra of SG (5 µM) alone at pH 8.3 (a) and pH 6.0 (b).

**Figure 5:**
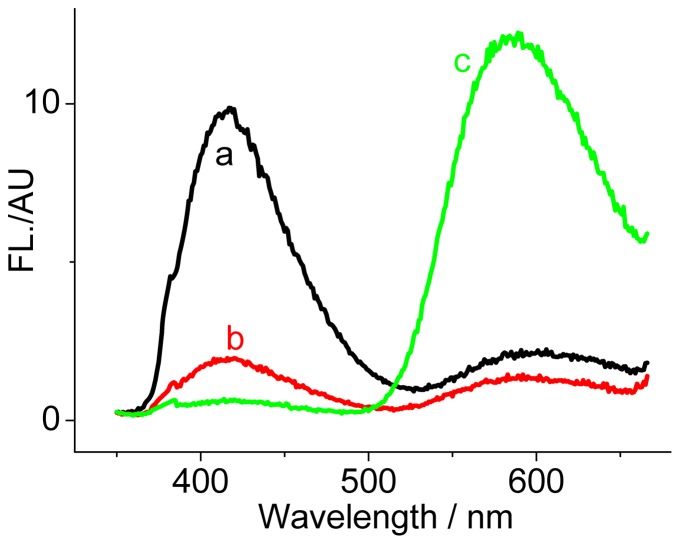
Emission spectra of SG (1 µM) in pH 8.3 aqueous solution. (a) SG alone; (b) in the presence of 5 µM FM-DNA; (c) after further addition of 1 µM DNA1-C with the SG solution pretreated with 5 µM FM-DNA.

From the absorption spectra (Figure 4A), besides the 336 nm absorption band, the presence of DNA1-Ys also increases the 405 nm and 470 nm absorption bands, as is occurred for the FM-DNA. This alteration in the absorption spectra was also observed for the other AP-DNAs (for example, DNA3-Ys, Figure S2). The 405 nm and 470 nm absorption bands result from the SG iminium form (Figure 4B) [33]. This phenomenon supports that the AP-DNAs as well as the FM-DNAs favor SG conversion from the alkanolamine form to the iminium form. Previously, Maiti et al. also reported that this conversion is possible when the concentration ratio of DNA nucleotide to SG is more than 6 [33]. In comparison to with the fluorescence behavior of SG bound to FM-DNA, the converted SG iminium form shows an enhancement in emission when bound to DNA1-Ys and DNA2-Ys and more quenching when bound to DNA3-Ys and DNA4-Ys, meaning that the SG iminium form is preferable to bind to the AP site. As an example in this aspect, we observed that the quenched fluorescence of 1 µM SG by 5 µM FM-DNA at 415 nm was bathochromically recovered at 586 nm only by further addition of 1 µM DNA1-T (Figure 5). No time-dependent spectral evolution was observed after thoroughly mixing DNA1-T and the FM-DNA-pretreated SG solution, indicating that the binding of SG to the AP site is very fast.

Relative to the AP site-dependent binding evidenced by the enhanced fluorescence responses for DNA1 and DNA2, the greater quenching for DNA3 and DNA4 with guanines and cytosines flanking the AP site does just mean that the SG binding behavior is really related to the presence of the AP site. The quenching should be caused by electron transfer between the excited-state SG bound at the AP site and the nearby guanines (G) because it is widely accepted that guanine is the most easily oxidizable base in DNA. Herein, the possibility of electron transfer was estimated by redox potentials of the involved species. The excited-state SG served as the electron acceptor with its reduction potential [43] E*_Red_ = E^0^_Red_+ΔE_0-0_. E^0^_Red_ was the reduction potential of the ground-state SG being about −0.56 V (vs. NHE) [44]. The singlet energy ΔE_0-0_ was calculated from the excitation (to be 493 nm from Figure 3, the excitation band very near the edge of the gap) and emission spectra of the bound SG, which was about 2.3 eV. Thus, the reduction potential E*_Red_ of the excited-state SG was calculated to be about 1.74 eV. However, the oxidation potentials (E^0^_Ox_) of nucleobases were reported to be about 1.47, 1.94, 2.09, and 2.12 V for guanine, adenine, thymine, and cytosine (vs. NHE), respectively [43]. Therefore, satisfying the condition of E*_Red_- E^0^_Ox_>0 should favor the occurrence of electron transfer between the AP site-bound excited-state SG as an electron acceptor and the nearby nucleobase as an electron donor. Clearly, only guanine is the case. Thus, for DNA1-Ys and DNA2-Ys with thymines and adenines flanking the AP site, the fluorescence enhancement was observed, while for DNA3-Ys with guanines flanking the AP site, the fluorescence quenching was observed. Although the AP site in DNA4-Ys is flanked by cytosines, not guanines, the guanines on the other strand paired with the flanking cytosines should also approach closely to the AP site-bound SG. Thus, the observed quenching for DNA4-Ys can be also explained by the electron transfer mechanism. The electron transfer rate should overwhelm the radiative decay rate for DNA3 and DNA4, which resulted in the observed fluorescence quenching. This electron transfer mechanism could be also employed to explain the lowest fluorescence enhancement that occurred for DNA1-G and DNA2-G in comparison to the corresponding AP-DNAs having the other unpaired bases. On the other hand, relative to purines, the small-sized pyrimidines opposite the AP site would provide more space in the AP site to effectively accommodate SG (see the following lifetime and T_m_ experiments), which should result in the observed slightly greater enhancements for DNAn-C and -T than DNAn-G and -A (n = 1, 2), and more quenching for DNAn-C and -T than DNAn-G and -A (n = 3, 4) (Figure 3 and S1).

**Figure 6:**
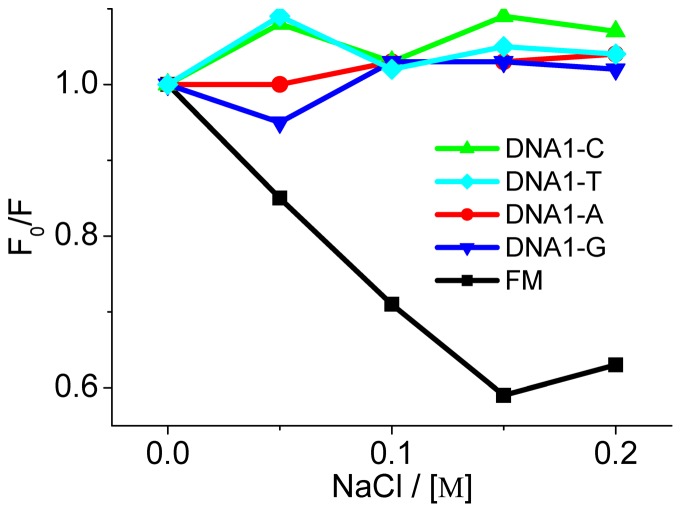
Effect of added NaCl concentrations on SG fluorescence at 586 nm. *F_0_* and *F* represent the fluorescence responses of the SG-DNA complexes in the absence and presence of NaCl, respectively.

In order to evaluate the SG binding mode, we checked the alterations in fluorescence upon adding the electrolyte of NaCl. As shown in Figure 6, addition of NaCl does not seriously affect the emission of SG bound to DNA1-Ys, whereas NaCl induces a concentration-dependent increase in fluorescence for the FM-DNA, indicating release of the bound SG from the FM-DNA upon increasing the Na^+^ concentration. These results confirm that the chromophore moiety of SG can mainly intercalate into the AP site. By contrast, a main minor groove binding of SG to FM-DNA [45] is expected because the minor groove site is the second strong Na^+^ binding site besides the phosphate backbone.

The fluorescence lifetime measurements were further used to evaluate the AP site binding of SG and the results were listed in Table 1. It is evidenced that the excited-state SG alone in aqueous solution decays according to a lifetime of 3.20 ns at 415 nm and of 2.45 ns at 586 nm for the alkanolamine form and iminium form, respectively, which is in good agreement with the previously reported values [46]. At 415 nm, the presence of FM-DNA, DNA1-A, and DNA1-G produces only one lifetime of 3.25, 3.32, and 3.30 ns respectively that is comparable with that for SG alone, showing that the alkanolamine form does not bind to these DNAs. The unfavorable binding of the alkanolamine form to FM-DNA has also been reported [37]. Nevertheless, besides the short-lived decays, both DNA1-C and -T induce another long-lived lifetime at this wavelength, implying that the alkanolamine form can bind to these AP sites. This could be explained by the fact that the small-sized pyrimidines opposite the AP site would provide more space in the AP site to effectively accommodate the more bulky SG alkanolamine nonplanar structure. Importantly, the increased average lifetimes for DNA1-C and -T (5.05 and 4.60 ns, in comparison to 3.20 ns for SG alone) and the increased excitation intensities at 336 nm (Figure 3A) would predict an enhanced emission at 415 nm. However, sharply decreased emissions were observed (Figure 3B), showing that a large population of the alkanolamine form converts to the iminium form. On the other hand, from the measured lifetimes at 586 nm (listed in Table 1), the SG iminium form is capable of binding to the FM-DNA and all DNA1-Ys. In comparison with a short-lived decay and a long-lived decay for DNA1-A and -G, only one long-lived decay was found for DNA1-C and -T, indicating a strong association of the iminium form to the AP site opposed by pyrimidines. For example, the intrinsic binding constants of 1.7×10^7^ M^−1^ and 8.3×10^5^ M^−1^ for DNA1-C and the FM-DNA respectively were derived from fluorescence titration experiments (Figure S3). The value for the FM-DNA without the AP site is in good agreement with the ones reported for natural and oligomeric DNAs [31]. Note that here only the binding modes related to the strongest DNA binding site for both DNA1-C and the FM-DNA were considered in calculating the corresponding binding parameters. Interestingly, the long-lived decay lifetimes of 14.05, 13.61, 12.05, and 11.75 ns for DNA1-C, -T, -A, and -G are just roughly proportional in turn to the oxidation potentials of their unpaired bases C, T, A, and G, again revealing that the bound SG' emission is somewhat affected by the possible electron transfer between the excited state SG and the unpaired bases opposite the AP site.

**Table 1:** Fluorescence decay fitting parameters (τ_1_ and τ_2_) of 5 µM SG in the absence and presence of 5 µM DNAsΔ.

	τ_1_ (ns)	τ_2_ (ns)	χ^2^
DNA free	3.20*^a^*		1.047
	2.45*^b^*		1.029
DNA1-A	3.32*^a^*		1.048
	2.90*^b^* (8.12%)	12.05 ^b^ (91.88%)	1.123
DNA1-C	2.76 ^a^ (74.27%)	11.65 ^a^ (25.73%)	1.003
		14.05 ^b^	1.032
DNA1-G	3.30 ^a^		1.067
	2.28 ^b^ (16.34%)	11.75 ^b^ (83.66%)	1.006
DNA1-T	2.87 ^a^ (76.62%)	10.29 ^a^ (23.38%)	1.019
		13.61 ^b^	1.039
FM-DNA	3.25 ^a^		1.063
	2.02 ^b^ (72.47%)	7.52 ^b^ (27.53%)	1.114

Δ The lifetimes were measured at 415 nm (a) and 586 nm (b) with excitation at 336 nm. The lifetime measurement was not applicable for DNA3-Ys and DNA4-Ys due to the strong fluorescence quenching.

From the above results, we can conclude that SG shows a sequence-dependent binding at the AP site. Usually, the specific interaction of small molecules with DNA base pairs will affect the DNA thermodynamic stability. In order to verify the occurrence of effective stacking interactions of SG with the AP-DNAs, DNA melting (T_m_) experiments were conducted by measuring the 260 nm absorbance as a function of the solution temperature. As shown in Table 2 to 5, the presence of SG stabilizes DNAn-C and DNAn-T with the T_m_ increasing of 4.4–5.4°C and 3.8–5.7°C, while DNAn-A and DNAn-G induce the T_m_ increasing of 1.9–3.2°C and 1.9–3.6°C, respectively. Thus, the small-sized pyrimidines opposite the AP site allow for an effective stacking interaction, which is predicted to result in the observed greater emission enhancements for DNAn-C and -T than DNAn-G and -A (n = 1, 2), and higher emission quenchings for DNAn-C and -T than DNAn-G and -A (n = 3, 4) (Figure 3 and S1). However, SG slightly stabilizes the FM-DNAs with the T_m_ increasing only of 0.5–0.8°C. Therefore, it is reasonably concluded that SG can enter into the hydrophobic helix interior in the presence of the AP site and the AP site is believed to play an important role for the occurrence of the stacking interaction, implying a binding mode different from that for the FM-DNAs. This effective π–π stacking interaction should efficiently prevent the converted SG from contacting with water and induce a pronounced fluorescence alteration, and thus favor the formation of the emissive iminium form when the AP site neighbors are bases other than guanines.

**Table 2:** Melting temperatures of 5 µM DNA1s in the absence and presence of 5 µM SG.

	FM1	DNA1-A	DNA1-C	DNA1-G	DNA1-T
With SG/°C	66.4	52.9	52.6	52.3	52.0
Without SG/°C	65.6	51.0	48.2	50.4	48.2
ΔT/°C	0.8	1.9	4.4	1.9	3.8

**Table 3:** Melting temperatures of 5 µM DNA2s in the absence and presence of 5 µM SG.

	FM2	DNA2-A	DNA2-C	DNA2-G	DNA2-T
With SG/°C	63.0	52.1	52.5	52.7	52.7
Without SG/°C	62.3	49.1	47.1	49.1	47.4
ΔT/°C	0.7	3.0	5.4	3.6	5.3

**Table 4:** Melting temperatures of 5 µM DNA3s in the absence and presence of 5 µM SG.

	FM3	DNA3-A	DNA3-C	DNA3-G	DNA3-T
With SG/°C	69.0	60.3	61.0	59.1	60.3
Without SG/°C	68.4	57.1	56.5	56.7	54.6
ΔT/°C	0.6	3.2	4.5	2.4	5.7

**Table 5:** Melting temperatures of 5 µM DNA4s in the absence and presence of 5 µM SG.

	FM4	DNA4-A	DNA4-C	DNA4-G	DNA4-T
With SG/°C	69.5	60.6	58.1	59.5	59.3
Without SG/°C	69.0	57.8	52.9	57.1	54.4
ΔT/°C	0.5	2.8	5.2	2.4	4.9

Finally, a comparison was made between SG binding to the AP site and to the mismatch site. With DNA1-C as an example, although we observed that the presence of a mismatch site (DNA1, X = T, Y = C, one of the most unstable mismatch sites [47]) also enhanced SG fluorescence, the enhancement arising from the AP site binding was still overwhelming (Figure S4), indicating the high selectivity of SG binding to the AP site. Additionally, a noticeable increase in the fluorescence response could be distinguished even with DNA1-C concentration as low as 500 nM when 5 µM SG was used. Thus, the AP site binding of SG could be probed with a high sensitivity.

## Conclusions

In summary, SG was found to serve as an effective AP site binder. However, its emission behavior is dependent on the sequences near the AP site. A sharp fluorescence enhancement for the iminium band and quenching for the alkanolamine band was observed for the DNAs having the AP site flanked by Ts and As. Thus, a large emission shift up to 170 nm is achieved. The emission enhancement is believed to be caused by the AP-site binding of the converted SG. Nevertheless, quenching of the two bands was observed only for the DNAs having the AP site flanked by Gs and Cs. The flanking G/C-induced quenching is likely to be caused by electron transfer between the AP site-bound SG in excited state and the nearby Gs. The featured emission properties of SG in the presence of the AP-DNAs are very promising to find applications in functional DNA-based biosensors with a large emission shift.

## Supporting Information

Figure S1 **AP site-dependent fluorescence behaviors of SG.** Excitation (A and C, measured at 586 nm), emission (B and D, excited at 336 nm) spectra of SG (5 µM) in the absence and presence of 5 µM DNA2-Ys (A and B) and DNA4-Ys (C and D). The corresponding fully matched DNAs (FM-DNA) were used as controls.
(TIF)

Figure S2 **Absorption spectra of SG-DNA3-Ys.** UV-Vis absorption spectra of SG (5 µM) in the absence and presence of 5 µM DNA3-Ys. The corresponding fully matched DNAs (FM-DNA) were used as controls.
(TIF)

Figure S3 **Scatchard plots for binding constant analysis.** Plots of *r*/*C*_f_ versus *r* for the interaction of SG (1 µM) with DNA1-C (A) and FM1 (B) by a fluorescence titration method on the basis of the Scatchard procedure. *r* is concentration of the bound SG per the added DNA concentration, and *C*_f_ free SG concentration. In order to simplify the calculations of *r* and *C*_f_, DNA1-C concentrations very near 1 µM were used to only get SG binding mainly at the AP site and avoid the simultaneous binding to the base pairs as occurred for FM-DNA, assuming that the AP site binding is much stronger than base pairs binding. The similar FM1 concentrations were employed for an effective comparison at the same concentration conditions. Binding constants of 1.7±0.15×10^7^ M^−1^ and 8.3±2.4×10^5^ M^−1^ for DNA1-C and FM1 were obtained, respectively. For accurate fluorescence determinations, the emission intensities at 586 nm for DNA1-C and that at 415 nm for FM1 were used for *r* and *C*_f_ calculations. Due to the stronger quenching than FM-DNA, the binding constants for DNA3-Ys or DNA4-Ys were not attempted.
(TIF)

Figure S4 **Comparison of SG's AP-site binding with a DNA-mismatch binding.** Fluorescence responses of 5 µM SG at 586 nm in the presence of DNA1-C, a mismatched DNA (DNA1, X = T, Y = C), and a fully matched DNA (DNA1, X = G, Y = C) with their concentrations at 0 nM, 50 nM, 100 nM, 500 nM, 1 µM, 3 µM, and 5 µM. Inset: the typical emission spectra at 5 µM of DNA.
(TIF)
