# Supplementary figures and images for: DNA Abasic Site-Selective Enhancement of Sanguinarine Fluorescence with a Large Emission Shift
Source: PLoS One. 2012 Nov 20;7(11):e48251. doi: 10.1371/journal.pone.0048251 (PMC3502418; doi:10.1371/journal.pone.0048251)

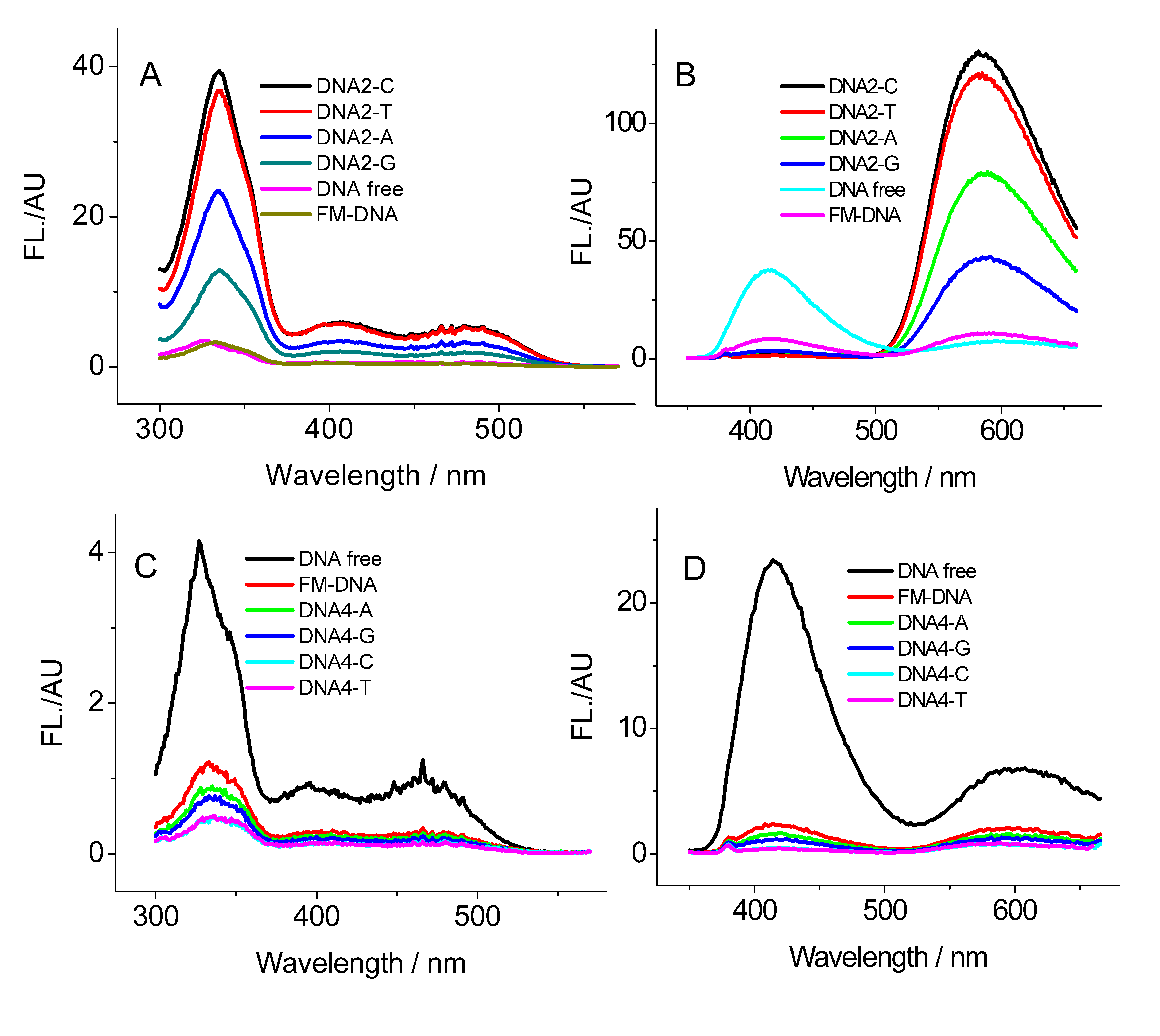

Supplement: Figure S1 — AP site-dependent fluorescence behaviors of SG. Excitation (A and C, measured at 586 nm), emission (B and D, excited at 336 nm) spectra of SG (5 µM) in the absence and presence of 5 µM DNA2-Ys (A and B) and DNA4-Ys (C and D). The corresponding fully matched DNAs (FM-DNA) were used as controls. (TIF) [file pone.0048251.s001.tif]

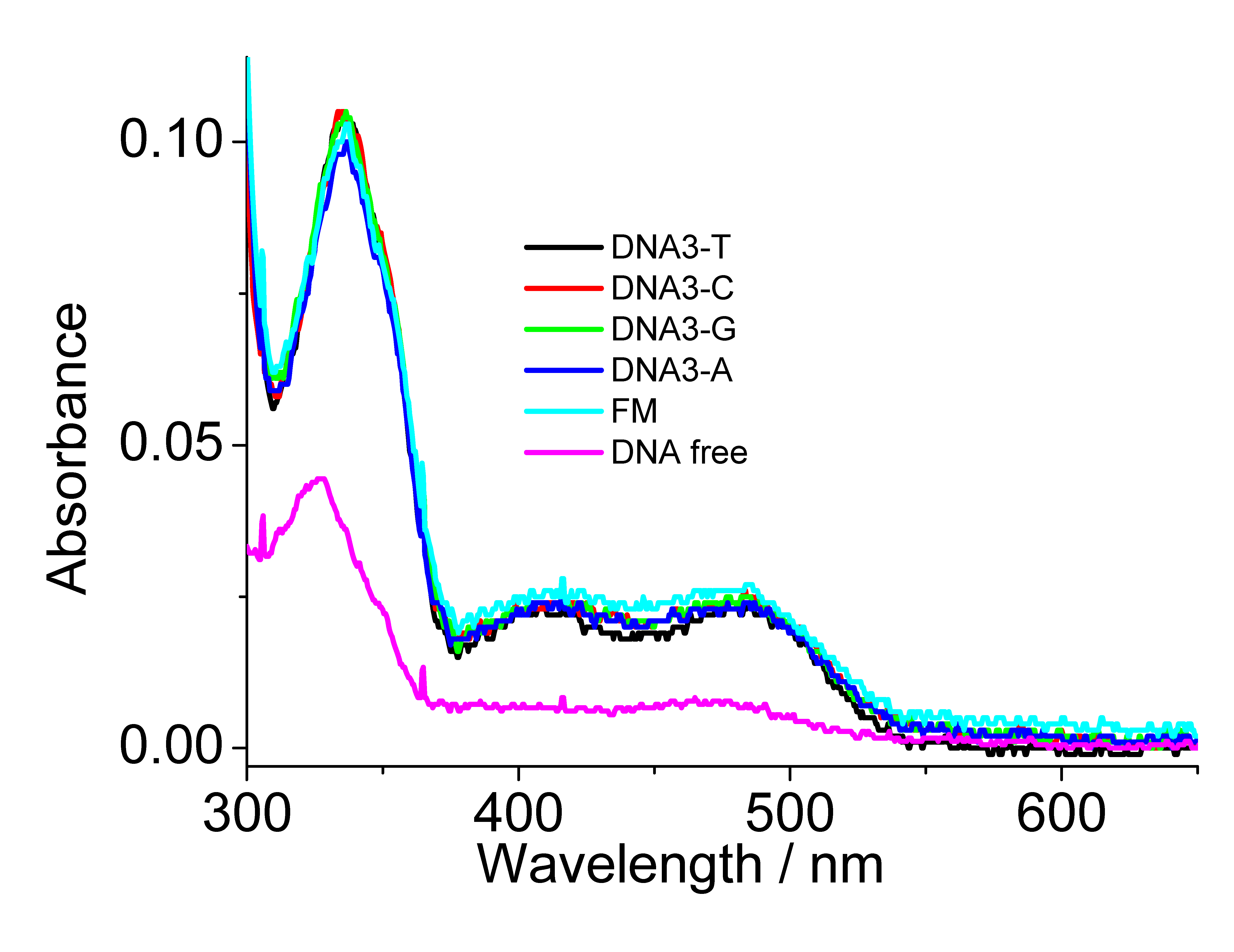

Supplement: Figure S2 — Absorption spectra of SG-DNA3-Ys. UV-Vis absorption spectra of SG (5 µM) in the absence and presence of 5 µM DNA3-Ys. The corresponding fully matched DNAs (FM-DNA) were used as controls. (TIF) [file pone.0048251.s002.tif]

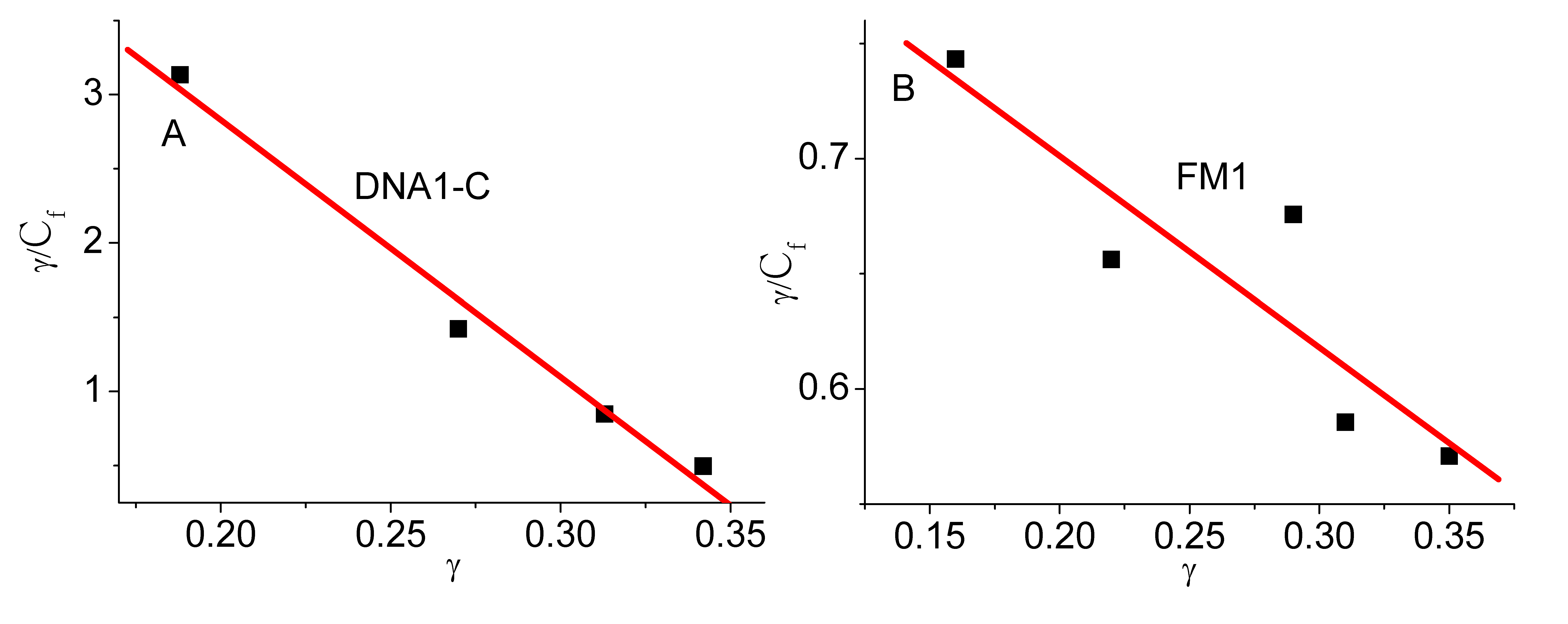

Supplement: Figure S3 — Scatchard plots for binding constant analysis. Plots of r/C f versus r for the interaction of SG (1 µM) with DNA1-C (A) and FM1 (B) by a fluorescence titration method on the basis of the Scatchard procedure. r is concentration of the bound SG per the added DNA concentration, and C f free SG concentration. In order to simplify the calculations of r and C f, DNA1-C concentrations very near 1 µM were used to only get SG binding mainly at the AP site and avoid the simultaneous binding to the base pairs as occurred for FM-DNA, assuming that the AP site binding is much stronger than base pairs binding. The similar FM1 concentrations were employed for an effective comparison at the same concentration conditions. Binding constants of 1.7±0.15×10^7^ M−1 and 8.3±2.4×10^5^ M−1 for DNA1-C and FM1 were obtained, respectively. For accurate fluorescence determinations, the emission intensities at 586 nm for DNA1-C and that at 415 nm for FM1 were used for r and C f calculations. Due to the stronger quenching than FM-DNA, the binding constants for DNA3-Ys or DNA4-Ys were not attempted. (TIF) [file pone.0048251.s003.tif]

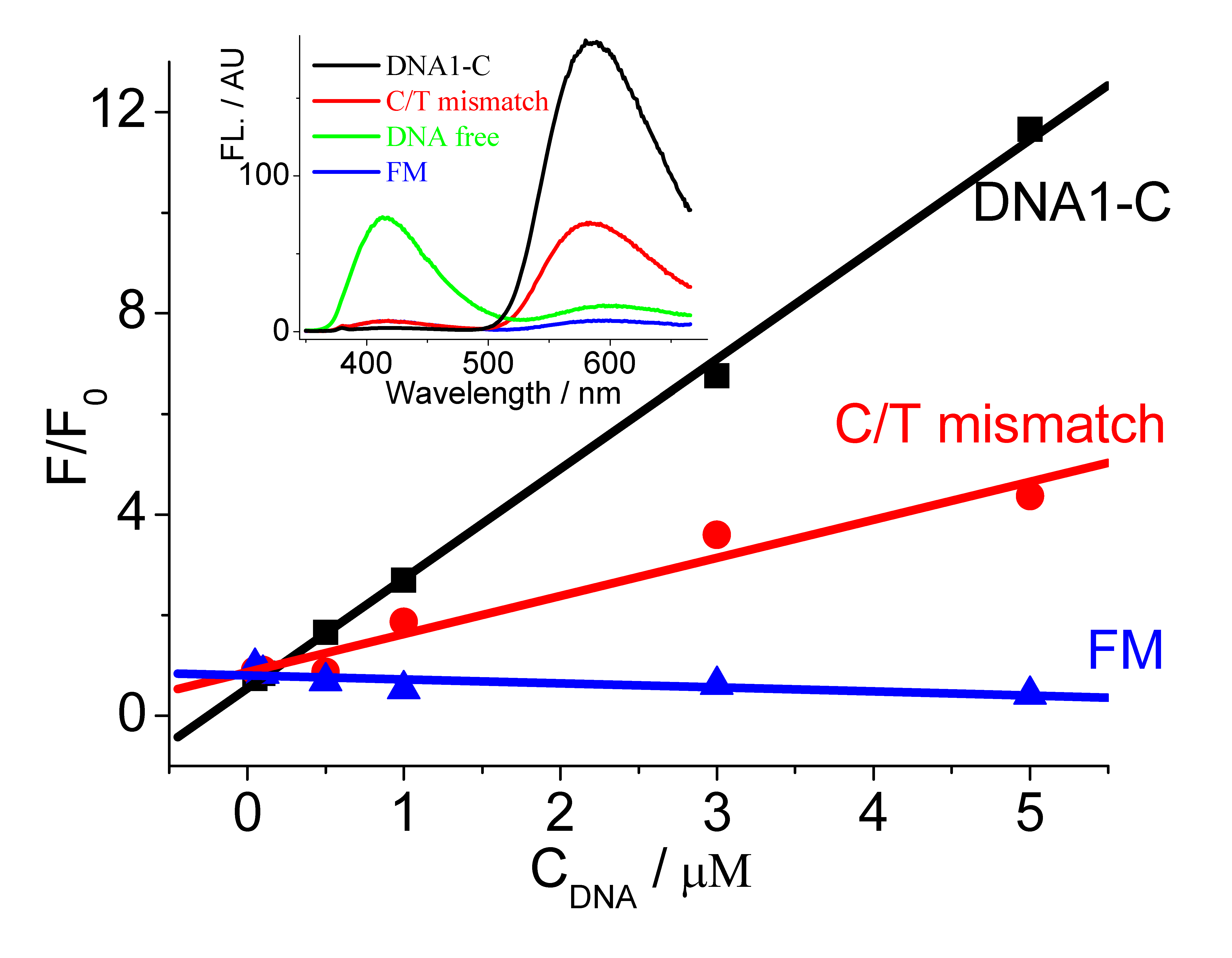

Supplement: Figure S4 — Comparison of SG's AP-site binding with a DNA-mismatch binding. Fluorescence responses of 5 µM SG at 586 nm in the presence of DNA1-C, a mismatched DNA (DNA1, X = T, Y = C), and a fully matched DNA (DNA1, X = G, Y = C) with their concentrations at 0 nM, 50 nM, 100 nM, 500 nM, 1 µM, 3 µM, and 5 µM. Inset: the typical emission spectra at 5 µM of DNA. (TIF) [file pone.0048251.s004.tif]
